# Visual Feedback of Tongue Movement for Novel Speech Sound Learning

**DOI:** 10.3389/fnhum.2015.00612

**Published:** 2015-11-19

**Authors:** William F. Katz, Sonya Mehta

**Affiliations:** Speech Production Lab, Callier Center for Communication Disorders, School of Behavioral and Brain Sciences, The University of Texas at DallasDallas, TX, USA

**Keywords:** speech production, second language learning, visual feedback, audiovisual integration, electromagnetic articulography, articulation therapy

## Abstract

Pronunciation training studies have yielded important information concerning the processing of audiovisual (AV) information. Second language (L2) learners show increased reliance on bottom-up, multimodal input for speech perception (compared to monolingual individuals). However, little is known about the role of viewing one's own speech articulation processes during speech training. The current study investigated whether real-time, visual feedback for tongue movement can improve a speaker's learning of non-native speech sounds. An interactive 3D tongue visualization system based on electromagnetic articulography (EMA) was used in a speech training experiment. Native speakers of American English produced a novel speech sound (/ɖ/; a voiced, coronal, palatal stop) before, during, and after trials in which they viewed their own speech movements using the 3D model. Talkers' productions were evaluated using kinematic (tongue-tip spatial positioning) and acoustic (burst spectra) measures. The results indicated a rapid gain in accuracy associated with visual feedback training. The findings are discussed with respect to neural models for multimodal speech processing.

## Introduction

Natural conversation is a multimodal process, where the visual information contained in a speaker's face plays an important role in decoding the speech signal. Integration of the auditory and visual modalities has long been known to be more advantageous to speech perception than either input alone. Early studies of lip-reading found that individuals with hearing loss could more accurately recognize familiar utterances when provided with both auditory and visual cues compared to either modality on its own (Numbers and Hudgins, 1948; Erber, 1975). Research on healthy hearing populations has also shown that audiovisual integration enhances comprehension of spoken stimuli, particularly in noisy environments or situations where the speaker has a strong foreign accent (O'Neill, 1954; Sumby and Pollack, 1954; Erber, 1975; Reisberg et al., 1987). Even under optimal listening conditions, observing a talker's face improves comprehension for complex utterances, suggesting that visual correlates of speech movement are a central component to processing speech sounds (Reisberg et al., 1987; Arnold and Hill, 2001).

Studies investigating how listeners process conflicting audio and visual signals also support a critical role of the visual system during speech perception (McGurk and MacDonald, 1976; Massaro, 1984; Summerfield and McGrath, 1984). For example, listeners presented with the auditory signal for “ba” concurrently with the visual signal for “ga” typically report a blended percept, the well-known “McGurk effect.” A recent study by Sams et al. (2005) demonstrated that the McGurk effect occurs even if the source of the visual input is the listener's *own* face. In this study, subjects wore headphones and silently articulated a “pa” or “ka” while observing their productions in a mirror as a congruent or incongruent audio stimulus was simultaneously presented. In addition to replicating the basic McGurk (blended) effect, researchers found that simultaneous silent articulation alone moderately improved auditory comprehension, suggesting that knowledge from one's own motor experience in speech production is also exploited during speech perception. Other cross-modal studies support this view. For instance, silently articulating a syllable in synchrony with the presentation of a concordant auditory and/or visually ambiguous speech stimulus has been found to improve syllable identification, with concurrent mouthing further speeding the perceptual processing of a concordant stimulus (Sato et al., 2013; also see Mochida et al., 2013; D'Ausilio et al., 2014). Taken together, these studies indicate that listeners benefit from multimodal speech information during the perception process.

Audiovisual (AV) information also plays an important role in acquiring novel speech sounds, according to studies of second language (L2) learning. Research has shown that speech comprehension by non-native speakers is influenced by the presence/absence of visual input (see Marian, 2009, for review). For instance, Spanish-speakers exposed to Catalan can better discriminate the non-native tense-lax vowel pair /e/ and /ε/ when visual information is added (Navarra and Soto-Faraco, 2007).

Computer-assisted pronunciation training (CAPT) systems have provided a new means of examining AV processing during language learning. Many CAPT systems, such as “Baldi” (Massaro and Cohen, 1998; Massaro, 2003; Massaro et al., 2006), “ARTUR” (Engwall et al., 2006; Engwall and Bälter, 2007; Engwall, 2008), “ATH” (Badin et al., 2008), “Vivian” (Fagel and Madany, 2008), and “Speech Tutor” (Kröger et al., 2010), employ animated talking heads, most of which can optionally display transparent vocal tracts showing tongue movement. “Tongue reading” studies based on these systems have shown small but consistent perceptual improvement when tongue movement information is added to the visual display. Such effects have been noted in word retrieval for acoustically degraded sentences (Wik and Engwall, 2008) and in a forced-choice consonant identification task (Badin et al., 2010).

Whereas the visual effects on speech perception are fairly well-established, the visual effects on speech production are less clearly understood. Massaro and Light (2003) investigated the effectiveness of using Baldi in teaching non-native phonetic contrasts (/r/-/l/) to Japanese learners of English. Both external and internal views (i.e., showing images of the speech articulators) of Baldi were found to be effective, with no added benefit noted for the internal articulatory view. A subsequent, rather preliminary report on English-speaking students learning Chinese and Arabic phonetic contrasts reported similar negative results for the addition of visual, articulatory information (Massaro et al., 2008). In this study, training with the Baldi avatar showing face (Mandarin) or internal articulatory processes (Arabic) provided no significant improvement in a small group of students' productions, as rated by native listeners.

In contrast, Liu et al. (2007) observed potentially positive effects of visual feedback on speech production for 101 English-speaking students learning Mandarin. This investigation contrasted three feedback conditions: audio only, human audiovisual, and a Baldi avatar showing visible articulators. Results indicated that all three methods improved students' pronunciation accuracy. However, for the final rime pronunciation both the human audiovisual and Baldi condition scores were higher than audio-only, with the Baldi condition significantly higher than the audio condition. This pattern is compatible with the view that information concerning the internal articulators helps relay information to assist in L2 production. Taken together, these studies suggest that adding visual articulatory information to 3D tutors can lead to improvements for producing certain language contrasts. However, more work is needed to establish the effectiveness, consistency, and strength of these techniques.

At the neurophysiological level, AV speech processing can be related to the issue of whether speech perception and production is supported by a joined action-observation matching system. Such a system has been related to “mirror” neurons originally described in the macaque brain [for reviews see (Rizzolatti and Craighero, 2004; Pulvermüller and Fadiga, 2010; Rizzolatti et al., 2014); although see (Hickok, 2009, 2010) for an opposing view]. Mirror neurons are thought to fire both during goal-directed actions and while watching a similar action made by another individual. Research has extended this finding to audiovisual systems in monkeys (Kohler et al., 2002) and speech processing in humans (e.g., Rizzolatti and Arbib, 1998; Arbib, 2005; Gentilucci and Corballis, 2006).

In support of this view, studies have linked auditory and/or visual speech perception with increased activity in brain areas involved in motor speech planning, execution, and proprioceptive control of the mouth (e.g., Möttönen et al., 2004; Wilson et al., 2004; Ojanen et al., 2005; Skipper et al., 2005, 2006, 2007a,b; Pekkola et al., 2006; Pulvermüller et al., 2006; Wilson and Iacoboni, 2006; Zaehle et al., 2008). Similarly, magnetoencephalography (MEG) studies have linked speech production with activity in brain areas specialized for auditory and/or visual speech perception processes (e.g., Curio et al., 2000; Gunji et al., 2001; Houde et al., 2002; Heinks-Maldonado et al., 2006; Tian and Poeppel, 2010). While auditory activation during speech production is expected (because acoustic input is normally present), Tian and Poeppel's (2010) study shows auditory cortex activation in the absence of auditory input. This suggests that an imaginary motor speech task can nevertheless generate forward predictions via an auditory efference copy.

Overall, these neurophysiological findings suggest a brain basis for the learning of speech motor patterns via visual input, which in turn would strengthen the multimodal speech representations in feedforward models. In everyday situations, visual articulatory input would normally be lip information only. However, instrumental methods of transducing tongue motion (e.g., magnetometry, ultrasound, MRI) raise the possibility that visual tongue information may also play a role.

Neurocomputational models of speech production provide a potentially useful framework for understanding the intricacies of AV speech processing. These models seek to provide an integrated explanation for speech processing, incorporated in testable artificial neural networks. Two prominent models include “Directions Into Velocities of Articulators” (DIVA) (Guenther and Perkell, 2004; Guenther, 2006; Guenther et al., 2006; Guenther and Vladusich, 2012) and “ACTion” (ACT) (Kröger et al., 2009). These models assume as input an abstract speech sound unit (a phoneme, syllable, or word), and generate as output both articulatory and auditory representations of speech. The systems operate by computing neural layers (or “maps”) as distributed activation patterns. Production of an utterance involves fine-tuning between speech sound maps, sensory maps, and motor maps, guided by feedforward (predictive) processes and concurrent feedback from the periphery. Learning in these models critically relies on forward and inverse processes, with the internal speech model iteratively strengthened by the interaction of feedback information.

Researchers have used neurocomputational frameworks to gain important insights about speech and language disorders, including apraxia of speech (AOS) in adults (Jacks, 2008; Maas et al., 2015), childhood apraxia (Terband et al., 2009; Terband and Maassen, 2010), developmental speech sound disorders (Terband et al., 2014a,b), and stuttering (Max et al., 2004; Civier et al., 2010). For example, DIVA simulations have been used to test the claim that apraxic disorders result from relatively preserved feedback (and impaired feed-forward) speech motor processes (Civier et al., 2010; see also Maas et al., 2015). These neurocomputational modeling-based findings correspond with largely positive results from visual augmented feedback intervention studies for individuals with AOS (see Katz and McNeil, 2010 for review; also, Preston and Leaman, 2014). Overall, these intervention findings have suggested that visual augmented feedback of tongue movement can help remediate speech errors in individuals with AOS, presumably by strengthening the internal model. Other clinical studies have reported that visual feedback can positively influence the speech of individuals with a variety of speech and language problems in children and adults, including articulation/phonological disorders, residual sound errors, and dysarthria. This research has included training with electropalatography (EPG) (Hardcastle et al., 1991; Dagenais, 1995; Goozee et al., 1999; Hartelius et al., 2005; Nordberg et al., 2011), ultrasound (Bernhardt et al., 2005; Preston et al., 2014) and strain gauge transducer systems (Shirahige et al., 2012; Yano et al., 2015).

Visual feedback training has also been used to study information processing during second language (L2) learning. For example, Levitt and Katz (2008) examined augmented visual feedback in the production of a non-native consonant sound. Two groups of adult monolingual American English speakers were trained to produce the Japanese post-alveolar flap /ɽ/. One group received traditional second language instruction alone and the other group received traditional second language instruction plus visual feedback for tongue movement provided by a 2D EMA system (Carstens AG100, Carstens Medizinelektronik GmbH, Bovenden, Germany, www.articulograph.de). The data were perceptually rated by monolingual Japanese native listeners and were also analyzed acoustically for flap consonant duration. The results indicated improved acquisition and maintenance by the participants who received traditional instruction plus EMA training. These findings suggest that visual information regarding consonant place of articulation can assist second language learners with accent reduction.

In another recent study, Suemitsu et al. (2013) tested a 2D EMA-based articulatory feedback approach to facilitate production of an unfamiliar English vowel (/æ/) by five native speakers of Japanese. Learner-specific vowel positions were computed for each participant and provided as feedback in the form of a multiple-sensor, mid-sagittal display. Acoustic analysis of subjects' productions indicated that acoustic and articulatory training resulted in significantly improved /æ/ productions. The results suggest feasibility and applicability to vowel production, although additional research will be needed to determine the separable roles of acoustic and articulatory feedback in this version of EMA training.

Recent research has shown that 3D articulography systems afford several advantages over 2D systems: recording in x/y/z dimensions (and two angles), increased accuracy, and the ability to track movement from multiple articulators placed at positions other than tongue midline (Berry, 2011; Kroos, 2012; Stella et al., 2013). As such, visual augmented feedback provided by these systems may offer new insights on information processing during speech production. A preliminary test of a 3D EMA-based articulatory feedback system was conducted by Katz et al. (2014). Monolingual English speakers were asked to produce several series of four CV syllables. Each series contained four different places of articulation, one of which was an alveolar (e.g., bilabial, velar, alveolar, palatal; such as /pa/-/ka/-/ta/-/ja/). A 1-cm target sphere was placed at each participant's alveolar region. Four of the five participants attempted the series with no visible feedback. The fifth subject was given articulatory visual feedback of their tongue movement and requested to “hit the target” during their series production. The results showed that subjects in the no-feedback condition ranged between 50 and 80% accuracy, while the subject given feedback showed 90% accuracy. These preliminary findings suggested that the 3D EMA system could successfully track lingual movement for consonant feedback purposes, and that feedback could be used by talkers to improve consonantal place of articulation during speech.

A more stringent test of whether 3D visual feedback can modify speech production would involve examining how individuals perform when they must achieve an unfamiliar articulatory target, such as a foreign speech sound. Therefore, in the present experiment we investigated the accuracy with which healthy monolingual talkers could produce a novel, non-English, speech sound (articulated by placing the tongue blade at the palatal region of the oral cavity) and whether this gesture could benefit from short-term articulatory training with visual feedback.

## Materials and methods

This study was conducted in accordance with the Department of Health and Human Services regulations for the protection of human research subjects, with written informed consent received from all subjects prior to the experiment. The protocol for this research was approved by the Institutional Review Board at the University of Texas at Dallas. Consent was obtained from all subjects appearing in audio, video, or figure content included in this article.

### Participants and stimuli

Five college-age subjects (three male, two female) with General American English (GAE) accents participated in this study. All talkers were native speakers of English with no speech, hearing, or language disorders. Three participants had elementary speaking proficiency with a foreign language (M03, F02: *Spanish*; F01: *French*). Participants were trained to produce a novel consonant in the /ɑCɑ/ context while an electromagnetic articulograph system recorded lingual movement. For this task, we selected a speech sound not attested as a phoneme among the world's languages: a voiced, coronal, palatal stop. Unlike palatal stops produced with the tongue body, found in languages such as Czech (/c/ and /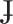/), subjects were asked to produce a closure with the tongue anterior (tip/blade) contacting the hard palate. This sound is similar to a voiced retroflex alveolar /ɖ/, but is articulated in the palatal, not immediately post-alveolar region. As such, it may be represented in the IPA as a backed, voiced retroflex stop: /ɖ/. Attested cases appear rarely in the world's languages and only as allophones. For instance, Dart (1991) notes some speakers of O'odham (Papago) produce voiced palatal sounds with (coronal) laminal articulation, instead of the more usual tongue body articulation (see Supplementary Materials for a sample sound file used in the present experiment).

Stimuli were elicited in blocks of 10 /ɑCɑ/ production attempts under a single-subject ABA design. Initially, the experimental protocol called for three pre-training, three training, and three post-training blocks from each subject (for a total of 90 productions). However, because data for this study were collected as part of a larger investigation of stop consonant productions, there was some subject attrition and reduced participation for the current experiment. Thus, the criterion for completion of the experiment was changed to a minimum of one block of baseline (no feedback) probes, 2–3 blocks of visual feedback training, and 1–3 blocks of post-feedback probes, for a total of 40–80 productions from each participant. All trials were conducted within a single experimental session lasting approximately 15 min.

### Procedure

Training sessions were conducted in a quiet testing room at the University of Texas at Dallas. Each participant was seated next to the Wave system, facing a computer monitor located approximately 1 m away. Five sensors were glued to the subject's tongue using a biocompatible adhesive: one each at tongue tip (~1 cm posterior to the apex), tongue middle (~3 cm posterior to apex), tongue back (~4 cm posterior to the apex), and both left and right tongue lateral positions. Sensors were also attached to a pair of glasses worn by the subject to establish a frame of reference for head movement. A single sensor was taped on the center of the chin to track jaw movement.

#### Visual feedback apparatus

**Figure 1:**
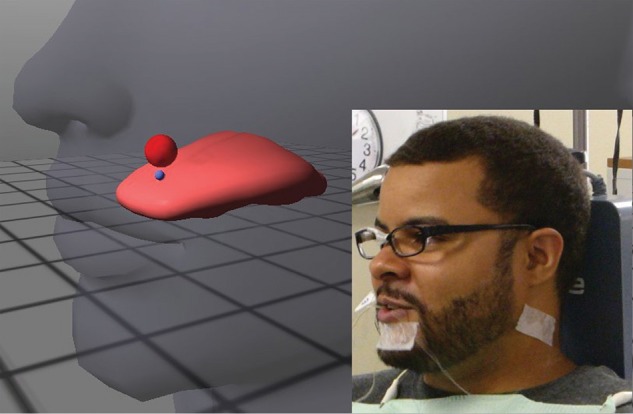
**Illustration of the ***Opti-Speech*** system, with subject wearing sensors and head-orientation glasses (lower right insert)**. A sample target sphere, placed in this example at the subject's alveolar ridge, is shown in red. A blue marker indicates the tongue tip/blade (TT) sensor.

External visual feedback for lingual movement was provided to subjects using a 3D EMA-based system (*Opti-Speech*, Vulintus LLC, Sachse, Texas, United States, http://www.vulintus.com/). This system works by tracking speech movement with a magnetometer (*Wave*, Northern Digital Incorporated, Waterloo, Ontario, Canada). An interface allows users to view their current tongue position (represented by an image consisting of flesh-point markers and a modeled tongue surface) within a transparent head with a moving jaw. Small blue spheres mark different regions on the animated tongue (tongue tip, tongue middle, tongue back, or tongue left/right lateral). Users may adjust the visibility of these individual markers and/or select or deselect “active” markers for speech training purposes. Articulatory targets, shown on the screen as semi-transparent red or orange spheres, can be placed by the user in the virtual oral cavity. The targets change color to green when the active marker enters, indicating correct tongue position, thus providing immediate visual feedback for place of articulation (see Katz et al., 2014 for more information). The target size and “hold time on target” can be varied by the user to make the target matching task easier or harder. An illustration of the system is shown in Figure 1.

#### Pronunciation training

The backed palatal stop consonant /ɖ/ is produced by making a closure between the tongue tip and hard palate. Therefore, the tongue tip marker was designated as the active marker for this study. A single target was placed at the palatal place of articulation to indicate where the point of maximum constriction should occur during the production of /ɖ/. To help set the target, participants were requested to press their tongue to the roof of their mouth, allowing the tongue sensors to conform to the contours of the palate. The experimenter then placed the virtual target at the location of the tongue middle sensor, which was estimated to correspond to the palatal (typically, pre-palatal) region. Based on previous work (Katz et al., 2014), we selected a target sphere of 1.00 cm in volume, with no hold time.

The current experiment was conducted as part of a larger study investigating stop consonant production that employed visual feedback for training purposes. As such, by the start of the experiment each participant had received an opportunity to accommodate to the presence of the Wave sensors on the tongue and to practice speaking English syllables and words under visual feedback conditions for approximately 25–30 min. In order to keep practice conditions uniform in the actual experiment, none of these warmup tasks involved producing a novel, non-English sound.

For the present experiment, participants were trained to produce the voiced, coronal, palatal stop, /ɖ/. The investigator (SM) described the sound to subjects as “sound[ing] like a ‘*d*,’ but produced further back in the mouth.” A more precise articulatory explanation was also provided, instructing participants to feel along the top of their mouth from front to back to help identify the alveolar ridge. Participants were then told to “place the tip of [their] tongue behind the alveolar ridge and slide it backwards to meet with the roof, or palate, of the mouth.” The investigator, a graduate student with a background in phonetics instruction, produced three repetitions of /ɑɖɑ/ (live) for participants to imitate. Each participant was allowed to practice making the novel consonantal sound 3–5 times before beginning the no-feedback trial sessions. This practice schedule was devised based on pilot data suggesting 3–5 practice attempts were sufficient for participants to combine the articulatory, modeled, and feedback information to produce a series of successive “best attempts” at the novel sound. Throughout the training procedure, the investigator provided generally encouraging comments. In addition, if an attempt was judged perceptually to be off-target (e.g., closer to an English /d/ or the palatalized alveolar stop, /d^j^/), the investigator pointed out the error and repeated the (articulatory) instructions.

**Figure 2:**
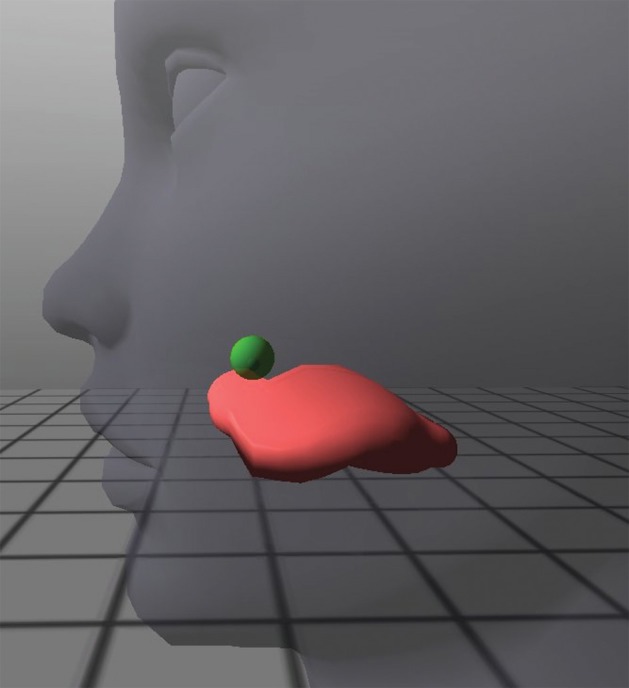
**Close-up of tongue avatar during a “hit” for the production of the voiced, retroflex, palatal stop consonant**. The target sphere lights up green, providing visual feedback for the correct place of articulation.

When the participant indicated that he/she understood all of the instructions, pre-training (baseline) trials began. After each block of attempts, participants were given general feedback about their performance and the instructions were reiterated if necessary. Once all pre-training sessions were completed, the participant was informed that the *Opti-Speech* visual feedback system would now be used to help them track their tongue movement. Subjects were instructed to use the tongue model as a guide for producing the palatal sound by moving the tongue tip upwards and backwards until the tongue tip marker entered the palatal region and the target lit up green, indicating success (see Figure 2). Each participant was allowed three practice attempts at producing the novel consonant while simultaneously watching the tongue model and aiming for the virtual target.

After completing the training sessions, the subject was asked to once again attempt to produce the sound with the visual feedback removed. No practice attempts were allowed between the training and post-training trial sessions. During all trials, the system recorded the talker's kinematic data, including a record of target hits (i.e., accuracy of the tongue-tip sensor entering the subject's palatal zone). The experiments were also audio- and video-recorded.

## Results

### Kinematic results

**Figure 3:**
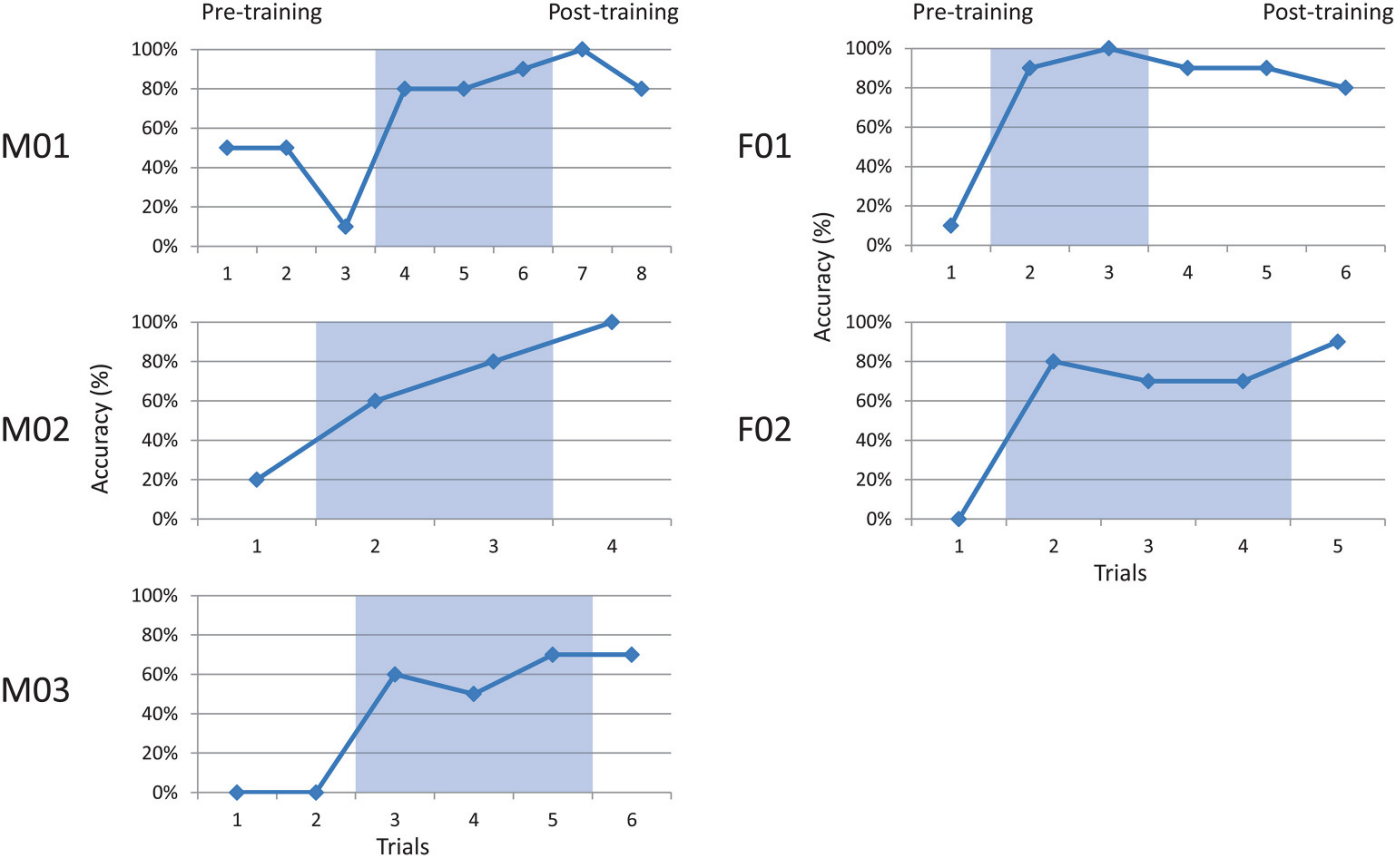
**Accuracy for five talkers producing a coronal palatal stop**. Shaded regions indicate visual feedback conditions. Baseline (pre-training) and post-training phases are also indicated.

All participants completed the speaking task without noticeable difficulty. Speakers' accuracy in achieving the correct articulation was measured as the number of hit targets out of the number of attempts in each block. Talker performance is summarized in Figure 3, which shows accuracy at the baseline (pre-training), visual feedback (shaded), and post-feedback (post-training) probes.

All talkers performed relatively poorly at baseline phase, ranging from 0 to 50% (*x* = 12.6%, *sd* = 14.1%) accuracy. Each participant showed a rapid increase in accuracy during the visual feedback phase (shaded), ranging from 50 to 100% (*x* = 74.9%, *sd* = 15.6). These gains appeared to be maintained during the post-feedback probes, with scores ranging from 70 to 100% (*x* = 85.3%, *sd* = 12.8%). Group patterns were examined using two-way paired *t*-tests. The results indicated a significant difference between pre-training and training phases, *t*_(4)_ = 8.73, *p* < 0.001, and pre-training and post-training phases, *t*_(4)_ = 14.0, *p* < 0.001. No significant difference was found between training and post-training, *t*_(4)_ = 1.66, *ns*. This pattern suggests acquisition during the training phase, and maintenance of learned behavior immediately post-training.

An effect size for each subject was computed using the Percentage of Non-overlapping Data (PND) method described by Scruggs et al. (1987). This non-parametric analysis compares points of non-overlap between baseline and successive intervention phases, and criteria are suggested for interpretation (Scruggs et al., 1986). Using this metric, all of the subjects' patterns were found to be greater than 90% (*highly effective*) for comparisons of both pre-training vs. training, and pre-training vs. post-training.

### Acoustic results

In order to corroborate training effects, we sought acoustic evidence of coronal (tongue blade) palatal stop integrity. This second analysis investigated whether the observed improvement in talkers' articulatory precision resulting from training would be reflected in patterns of the consonant burst spectra. Short-term spectral analyses were obtained at the moment of burst release (Stevens and Blumstein, 1975, 1978). Although, burst spectra may vary considerably from speaker to speaker, certain general patterns may be noted. Coronals generally have energy distribution across the whole spectrum, with at least two peaks between 1.2 and 3.6 kHz), termed “diffuse” in the feature system of Jakobson et al. (1952). Also, coronals typically result in relatively higher-frequency spectral components than articulations produced by lips or the tongue body, and these spectra are therefore described as being “acute” (Jakobson et al., 1952; Hamann, 2003) or “diffuse-rising” (Stevens and Blumstein, 1978).

Burst frequencies vary as a function of the length of the vocal tract anterior to the constriction. Thus, alveolar constriction results in a relatively high burst, ranging from approximately 2.5 to 4.5 kHz (e.g., Reetz and Jongman, 2009), while velar stops, having a longer vocal tract anterior to the constriction, produce lower burst frequencies (ranging from approximately 1.5 to 2.5 kHz). Since palatal stops are produced with a constriction located between the alveolar and velar regions, palatal stop bursts may be expected to have regions of spectral prominence between the two ranges, in the 3.0–5.0 kHz span. Acoustic analyses of Czech or Hungarian velar and palatal stops generally support this view. For instance, Keating and Lahiri (1993) note that the Hungarian palatal stop /ca/ spectrum slopes up to its highest peak “at 3.0–4.0 kHz or ever higher,” but otherwise show “a few peaks of similar amplitude which together dominate the spectrum in a single broad region” (p. 97). A study by Dart (1991) obtained palatographic and spectral data for O'odham (Papago) voiced palatal sounds produced with laminal articulation. Analysis of the burst spectra for these (O'odham) productions revealed mostly diffuse rising spectra, with some talkers showing “a high amplitude peak around 3.0–5.0 Hz” (p. 142).

For the present experiment, three predictions were made: (1) palatal stop consonant bursts prior to training will have diffuse rising spectra with characteristic peaks in the 3.0–5.0 kHz range, and (2) following training, these spectral peaks will shift downwards, reflecting a more posterior constriction (e.g., from an alveolar toward a palatal place of articulation), and (3) post-training token-to-token variability should be lower than at baseline, reflecting increased articulatory ability.

### Spectral analysis

Talkers' consonantal productions were digitized and analyzed using PRAAT (Boersma and Weenink, 2001) with a scripting procedure using linear predictive coding (LPC) analysis. A cursor was placed at the beginning of the consonant burst of each syllable and a 12 ms Kaiser window was centered over the stop transient. Autocorrelation-based LPC (24 pole model, +6 dB pre-emphasis) yielded spectral sections. Overlapping plots of subjects' repeat utterances were obtained for visual inspection, with spectral peaks recorded for analysis.

**Figure 4:**
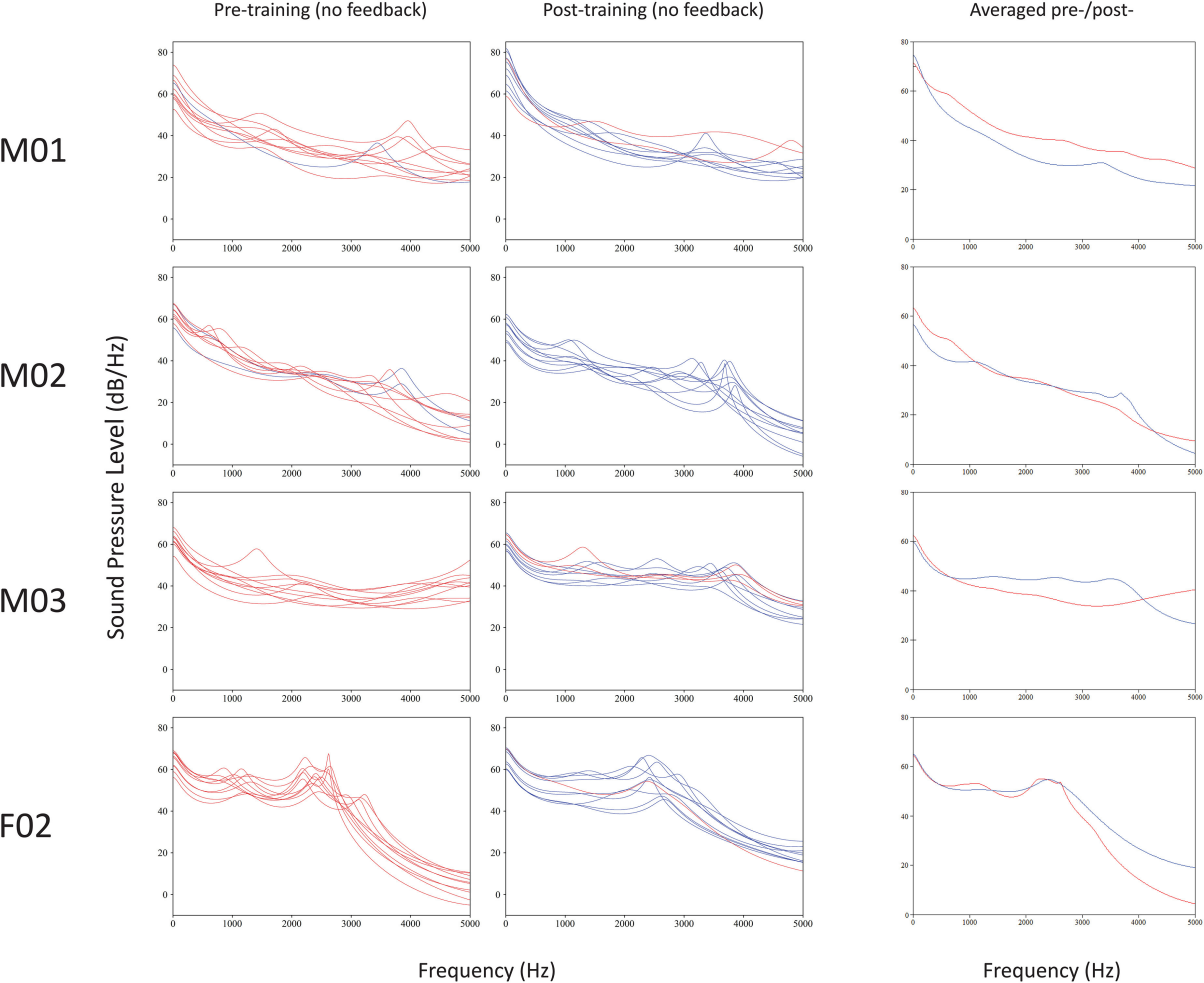
**Overlapping plots of short-term spectra for bursts of voiced, coronal, palatal stops produced before and after EMA training**. Correct place of articulation (hits) are marked in blue, and errors (misses) in red. Computed averages of incorrect pre-training (red) and correct post-training (blue) spectra are shown at right, for comparison.

Figure 4 shows overlapping plots of spectra obtained pre- and post-EMA training for 4/5 talkers. Plots containing (RMS) averages for pre-training (incorrect) and post-training (correct) spectra are also shown, for comparison. Spectra for talker F01 could not be compared because this talker's initial productions were realized as CV syllables (instead of VCV), and differing vowel context is known to greatly affect burst consonant spectral characteristics (Stevens, 2008).

Results revealed mixed support for the experimental predictions. Similar to previous reports (e.g., Dart, 1991), there were considerable differences in the shapes of the burst spectral patterns from talker to talker. Three of the four talkers' spectra (M01, M02, and M03) were diffuse, having at least two peaks between 1.2 and 3.6 kHz, while the spectra of talker F01 had peaks in a mid-frequency (“compact”) range of 2.0–3.0 kHz. Patterns of spectral tilt for all speakers were generally falling (instead of rising, as expected).

The prediction that 3.0–5.0 kHz spectral peak frequencies would lower following training was not uniformly obtained. Because standard deviations were relatively high and there was much inter-talker variability, the data are summarized, rather than tested statistically.

Talker M01's data had six peaks pre-treatment (*x* = 3967; *sd* = 596) and five peaks post-training (*x* = 4575; *sd* = 281). Talker M02's productions yielded five peaks pre-training (*x* = 3846; *sd* = 473) and nine peaks post-training (*x* = 3620; *sd* = 265). Talker M03 had six peaks pre-training (*x* = 4495 *sd* = 353) and nine peaks post-training (*x* = 3687; *sd* = 226). The spectra of talker F02 had peaks in a mid-frequency (“compact”) range of approximately 2.0–3.0 kHz. This talker's spectral peak values did not shift with training (pre-training: *x* = 2359 Hz, *sd* = 139 Hz; post-training: *x* = 2390 Hz, *sd* = 194 Hz). In summary, talkers M03 and M02 showed the expected pattern of spectra peak lowering, F02 showed no training-dependent changes, and M01 showed a pattern in the opposite direction.

Of the talkers with spectra data available, three (M01, M02, and M03) showed marked reduction in variability (i.e., reduced standard deviation values) from pre-training to post-training, suggesting that training corresponded with increased production consistency. However, this was not the case for talker F02, whose mid-range spectral peaks showed a slight increase in variability after training.

## Discussion

Five English-speaking subjects learned a novel consonant (a voiced, coronal, and palatal stop) following a brief training technique involving visual augmented feedback of tongue movement. The results of kinematic analyses indicate that real-time visual (articulatory) feedback resulted in improved accuracy of consonant place of articulation. Articulatory feedback training for place of articulation corresponded with a rapid increase in the accuracy of tongue tip spatial positioning, and post-training probes indicated (short-term) retention of learned skills.

Acoustic data for talkers' burst spectra obtained pre- and post-training only partially confirmed the kinematic findings, and there were a number of differences noted from predictions. First, for those talkers that showed diffuse spectra (e.g., with two peaks between 1.2 and 3.6 kHz), the spectra were falling, instead of rising. This may have been due to a number of possible factors, including the current choice of a Kaiser window for spectral analysis. Some of the original studies, such as those which first noted the classic “diffuse rising” patterns in spectral slices, fitted half-Hamming windows over the burst to obtain optimum pre-emphasis for LPC analysis (e.g., Stevens and Blumstein, 1978). Second, talker F02 showed mid-range (“compact”) spectral peaks ranging between 2.0 and 3.0 kHz. This may be due to tongue shape, which can affect the affect spectral characteristics of the stop burst. For example, laminal (tongue blade) articulation results in relatively even spectral spread, while apical (tongue-tip) articulation results in strong mid-frequency peaks (Ladefoged and Maddieson, 1996) and less spread (Fant, 1973). In the present data, the spectra of talker F02 fits that pattern of a more apical production.

Despite individual differences, there was some evidence supporting the notion of training effects in the acoustic data. Chiefly, the three subjects with diffuse spectra (M01, M02, and M03) showed decreased variability (lowered standard deviations) following training, suggesting stabilized articulatory behavior. Although the current data are few, they suggest that burst spectra variability may be a useful metric to be explored in future studies.

It was predicted that spectral peaks in the 3.0–5.0 kHz range would lower in frequency as talkers improved their place of articulation, with training. However, the findings do not generally support this prediction: Talker M03 showed this pattern, M02 showed a trend, F02 showed no differences, and M01 trended in the opposite direction, with higher spectral peaks after training. Since the kinematic data establish that all talkers significantly increased tongue placement accuracy post-training, we speculate that several factors affecting burst spectra (e.g., tongue shape, background noise, or room acoustics) may have obscured any such underlying spectral shifts for the talkers. Future research should examine how burst spectra may be best used to evaluate outcomes in speech training studies.

The current kinematic data replicate and extend the findings of Ouni (2013) who found that talkers produced tongue body gestures more accurately after being exposed to a short training session of real-time ultrasound feedback (post-test) than when recorded at baseline (pre-test). The present results are also consistent with earlier work from our laboratory which found that monolingual English speakers showed faster and more effective learning of the Japanese post-alveolar flap, /ɽ/ using EMA-based visual feedback, when compared with traditional Japanese pronunciation instruction (Levitt and Katz, 2008). Taken together with the experimental data from this study, there is evidence that EMA-provided articulatory visual feedback may provide a means for helping L2 learners improve novel consonant distinctions.

However, a number of caveats must be considered. First, the current data are limited and the study should therefore be considered preliminary. The number of subjects tested was few (*n* = 5). Also, since the consonant trained, /ɖ/, is not a phoneme in any of the world's language, it was not possible to include perceptual data, such as native listener judgments (e.g., Levitt and Katz, 2010). Additional data obtained from more talkers will therefore be required before any firm conclusions can be drawn concerning the relation to natural language pronunciation.

Second, real-time (live) examples were given to subjects by the experimenter (SM) during the training phase, allowing for the possibility of experimenter bias. This procedure was adopted to simulate a typical second-language instruction setting, and care was taken to produce consistent examples, so as to not introduce “unfair” variability at the start of the experiment. Nevertheless, in retrospect it would have been optimal to have included a condition in which talkers were trained with pre-recorded examples, to eliminate this potential bias.

Third, since articulatory training is assumed to draw on principles of motor learning, several experimental factors must be controlled before it is possible to conclude that a given intervention is optimal for a skill being acquired, generalized, or maintained (e.g., Maas et al., 2008; Bislick et al., 2012; Schmidt and Lee, 2013; Sigrist et al., 2013). For example, Ballard et al. (2012) conducted a study in which a group of English talkers was taught the Russian trilled /r/ sound using an EPG-based visual feedback system. In a short-term (five session) learning paradigm, subjects practiced in conditions either with continuous visual feedback provided by an EPG system, or were given no visual feedback. The results suggested that providing kinematic feedback continually though treatment corresponded with lower skill retention. This finding suggests that speech training follows the principle that kinematic feedback is most beneficial in the early phases of training, but may interfere with long-term retention if provided throughout training (Swinnen et al., 1993; Hodges and Franks, 2001; Schmidt and Lee, 2013). A pattern in the current data also potentially supports this principle. Three of the five participants (M01, M03, and F02) reached their maximum performance in the post-training phase, immediately after the feedback was removed. While this pattern was not statistically significant, it may suggest some interference effects from the ongoing feedback used. Future research should examine factors such as feedback type and frequency in order to better improve speech sound learning.

**Figure 5:**
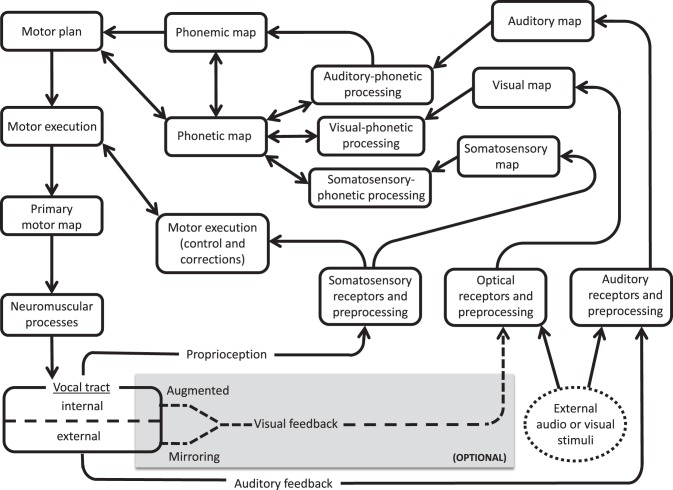
**Simplified version of ACT model (Kröger and Kannampuzha, 2008), showing input pathways for external audiovisual stimuli (oval at bottom right) and optional feedback circuits to the vocal tract (shaded box at bottom)**. Visual feedback (dotted line) is provided by either external (mirroring) or internal (instrumental augmented) routes.

The current findings support the notion of a visual feedback pathway during speech processing, as proposed in the ACT neurocomputational model of speech production (Kröger and Kannampuzha, 2008). Similar to the DIVA model, ACT relies on feedforward and feedback pathways between distributed neural activation patterns, or maps. ACT includes explicit provisions for separate visual and auditory information processing. In Figure 5, we present a simplified model of ACT (adapted from Kröger et al., 2009) with (optional) modifications added to highlight pathways for external and internal audiovisual input. Since people do not ordinarily rely on visual feedback of tongue movement, these modifications explain how people learn under conditions of augmented feedback, rather than serving as key components of everyday speech.

The external input route (dotted circle on the right) indicates an outside speech source, including speech that is produced while hearing/observing human talkers or a computerized training agent (e.g., BALDI, ARTUR, ATH, or Vivian). The input audio and visual data are received, preprocessed, and relayed as input to respective unimodal maps. These maps yield output to a multimodal phonetic map that also receives (as input) information from a somatosensory map and from a phonemic map. Reciprocal feedback connections between the phonetic map, visual-phonetic processing, and auditory-phonetic processing modules can account for training effects from computerized training avatars. These pathways would presumably also be involved in AV model-learning behavior, including lip-reading abilities (see Bernstein and Liebenthal, 2014 for review) and compensatory tendencies noted in individuals with left-hemisphere brain damage, who appear to benefit from visual entrainment to talking mouths other than their own (Fridriksson et al., 2012).

In the (internal) visual feedback route (dotted arrows), a talker's own speech articulation is observed during production. This may include simple mirroring of the lips and jaw, or instrumentally augmented visualizations of the tongue (via EMA, ultrasound, MRI, or articulatory inversion systems that convert sound signals to visual images of the articulators; e.g., Hueber et al., 2012). The remaining audio and visual preprocessing and mapping stages are similar between this internal route and the external (modeled) pathways. The present findings of improved consonantal place of articulation under conditions of visual (self) feedback training supports this internal route and the role of body sense/motor familiarity. This internal route may also play a role in explaining a number of other phenomena described in the literature, including the fact that talkers can discern between natural and unnatural tongue movements displayed by an avatar (Engwall and Wik, 2009), and that training systems based on a talkers' own speech may be especially beneficial for L2 learners (see Felps et al., 2009 for discussion).

The actual neurophysiological mechanisms underlying AV learning and feedback are currently being investigated. Recent work on oral somatosensory awareness suggests people have a unified “mouth image” that may be qualitatively different from other parts of the body (Haggard and de Boer, 2014). Since visual feedback does not ordinarily play a role in mouth experiences, other attributes, such as self-touch, may play a heightened role. For instance, Engelen et al. (2002) note that subjects can achieve high accuracy in determining the size of ball-bearings placed in the mouth, but show reduced performance when fitted with a plastic palate. This suggests that relative movement of an object between tongue and palate is important in oral size perception. We speculate that visual feedback systems rely in part on oral self-touch mechanism (particularly for consonant production), by visually guiding participants to the correct place of articulation, at which point somatosensory processes take over. This mechanism may prove particularly important for consonants, as opposed to vowels, which are produced with less articulatory contact.

Providing real-time motor feedback may engage different cortical pathways than are recruited in learning systems that employ more traditional methodologies. For example, Farrer et al. (2003) conducted positron emission tomography (PET) experiments in which subjects controlled a virtual hand on a screen under conditions ranging from full control, to partial control, to a condition where another person controlled the hand and there was no control. The results showed right inferior parietal lobule activation when subjects felt least in control of the hand, with reverse covariation in the insula. A crucial aspect here is corporeal identity, the feeling of one' own body, in order to determine motor behavior in the environment. Data suggest that body awareness is supported by a large network of neurological structures including parietal and insular cortex, with primary and secondary somatosensory cortex, insula, and posterior parietal cortex playing specific roles (see Daprati et al., 2010 for review). A region of particular interest is the right inferior parietal lobule (IPL), often associated to own-body perception and other body discrimination (Berlucchi and Aglioti, 1997; Farrer et al., 2003; Uddin et al., 2006). Additional neural structures that likely play a role in augmented feedback training systems include those associated with reward dependence during behavioral performance, including lateral prefrontal cortex (Pochon et al., 2002; Liu et al., 2011; Dayan et al., 2014). As behavioral data accrue with respect to both external (mirroring) and internal (“tongue reading”) visual speech feedback, it will be important to also describe the relevant neural control structures, in order to best develop more complete models of speech production.

In summary, we have presented small-scale but promising results from an EMA-based feedback investigation suggesting that augmented visual information concerning one's own tongue movements boosts skill acquisition during the learning of consonant place of articulation. Taken together with other recent data (e.g., Levitt and Katz, 2010; Ouni, 2013; Suemitsu et al., 2013) the results may have potentially important implications for models of speech production. Specifically, distinct AV learning mechanisms (and likely, underlying neural substrates) appear to be engaged for different types of CAPT systems, with interactive, on-line, eye-to-tongue coordination involved in systems such as *Opti-Speech* (and perhaps *Vizart3D*, Hueber et al., 2012) being arguably different than processing involved in using external avatar trainers, such as ARTUR, BALDI, ATH, or Vivian. These different processing routes may be important when interpreting other data, such as the results of real-time, discordant, cross-modal feedback (e.g., McGurk effect). Future, studies should focus on extending the range of speech sounds, features, and articulatory structures trained with real-time feedback, with a focus on vowels as well as consonants (see Mehta and Katz, 2015). As findings are strengthened with designs that systematically test motor training principles, the results may open new avenues for understanding how AV information is used in speech processing.

## Author contributions

WK and SM designed the experiments. SM recruited the participants and collected the data. WK and SM performed the kinematic analysis. WK conducted the spectral analysis. WK and SM wrote the manuscript.

### Conflict of interest statement

This research was partially supported by a grant to Vulintus, LLC entitled “Development of a software package for speech therapy” (NIH-SBIR 1 R43 DC013467). However, the sources of support for this work had no role in the study design, collection, analysis or interpretation of data, or the decision to submit this report for publication. The corresponding author (William F. Katz) had full access to all of the data in the study and takes complete responsibility for the integrity and accuracy of the data. The authors declare that the research was conducted in the absence of any commercial or financial relationships that could be construed as a potential conflict of interest.
